# Measuring sleep quality in older adults: a comparison using subjective and objective methods

**DOI:** 10.3389/fnagi.2015.00166

**Published:** 2015-09-07

**Authors:** Glenn J. Landry, John R. Best, Teresa Liu-Ambrose

**Affiliations:** ^1^Department of Physical Therapy, University of British ColumbiaVancouver, BC, Canada; ^2^Djavad Mowafaghian Centre for Brain Health, University of British ColumbiaVancouver, BC, Canada; ^3^Brain Research Centre, University of British ColumbiaVancouver, BC, Canada

**Keywords:** circadian, sleep, aging, actigraphy, Pittsburg Sleep Quality Index, Consensus Sleep Diary, mild cognitive impairment, dementia

## Abstract

Sleep quality decreases with aging and thus sleep complaints are prevalent in older adults, particularly for those with cognitive impairment and dementia. For older adults, emerging evidence suggests poor sleep quality increases risk of developing cognitive impairment and dementia. Given the aging population—and the impending economic burden associated with increasing numbers of dementia patients—there is pressing need to improve sleep quality among older adults. As such, research efforts have increased focus on investigating the association between age-related sleep changes and cognitive decline in older adults. Sleep quality is a complex construct to evaluate empirically, and yet the Pittsburg Sleep Quality Index (PSQI) is commonly used in studies as their only measure of sleep quality. Furthermore, the PSQI may not be the best sleep quality measure for older adults, due to its reliance on the cognitive capacity to reflect on the past month. Further study is needed to determine the PSQI's validity among older adults. Thus, the current study examined sleep quality for 78 community dwelling adults 55+ to determine the PSQI's predictive validity for objective sleep quality (as measured by actigraphy). We compared two subjective measures of sleep quality—the PSQI and Consensus Sleep Diary (CSD)—with actigraphy (MotionWatch 8©; cam*n*tech). Our results suggest perceived sleep quality is quite different from objective reality, at least for adults 55+. Importantly, we show this difference is unrelated to age, gender, education, or cognitive status (assessed using standard screens). Previous studies have shown the PSQI to be a valuable tool for assessing subjective sleep quality; however, our findings indicate for older adults the PSQI should not be used as a substitute for actigraphy, or vice versa. Hence, we conclude best practice is to include both subjective and objective measures when examining sleep quality in older adults (i.e., the PSQI, CSD, and actigraphy).

## Introduction

Sleep quality changes as a function of normal aging, both in terms of decreased duration and consolidation (for reviews see Espiritu, 2008; Crowley, 2011). Recent findings suggest sleep quality plays a critical role in preserving cognitive function in older adults and reducing the risk of dementia (Lim et al., 2013). Unfortunately, sleep complaints are common among older adults—more than half of adults 65+ have at least one chronic sleep complaint—the most common being an inability to stay asleep at night (Foley et al., 1995). Given the world's aging population, understanding how changes in sleep quality may contribute to cognitive decline among older adults has become a research imperative (reviewed in Landry and Liu-Ambrose, 2014). However, sleep quality is a complex construct, making it difficult to evaluate empirically. As such, the validity of current and future research efforts depends greatly on the methods used to quantify parameters of sleep quality. Historically, sleep quality has been assessed using various methods, including subjective measures (e.g., the Consensus Sleep Diary and Pittsburgh Sleep Quality Index) and objective measures (e.g., polysomnography and actigraphy).

The Consensus Sleep Diary (CSD; Carney et al., 2012) is the product of collaborations with insomnia experts and potential users. This workgroup designed, tested, and refined a consensus-based standardized sleep diary to be used primarily for the purposes of insomnia research, but also for clinical and research applications for both “good” and “poor” sleepers. In the current study we used the CSD-Core—a 9-item diary—considered by the CSD workgroup and their focus group participants to represent the most critical sleep parameters.

The Pittsburgh Sleep Quality Index (PSQI; Buysse et al., 1989) was originally developed to provide clinicians with a valid, standardized measure of sleep quality that could reliably categorize individuals as either “good” or “poor” sleepers. This 19-item questionnaire assesses sleep quality using subjective ratings for 7 different components (i.e., sleep quality; sleep latency; sleep duration; habitual sleep efficiency; sleep disturbance; use of sleeping medication; and daytime dysfunction). Respondents are asked to answer the questionnaire retrospectively, surveying sleep components spanning the previous month. The PSQI is quick and easy to administer, and score; making it an attractive tool for sleep quality assessments.

Since its introduction the PSQI has emerged as the de facto gold standard subjective measure of sleep quality. However, as Buysse et al. (1989) explained, the PSQI does not correlate well with polysomnography (PSG). Yet, PSG is the gold standard objective measure of sleep: it provides the most accurate assessment of sleep quality, quantity, and architecture (Littner et al., 2003a). Buysse et al. (1989) suggested the retrospective nature of the PSQI—a global sleep quality estimate spanning the previous month—could explain its limited agreement with single night PSG recordings. Perhaps PSG recordings averaged over the same period queried by the PSQI would be correlated, but this assertion needs to be confirmed empirically. However, the invasive nature of PSG—usually requiring an overnight stay in a sleep laboratory or clinic—makes long-term multi-night recordings impractical.

Fortunately, technological advances have led to the development of battery powered, long-life, light-weight, non-invasive, wearable accelerometers measuring tri-axial movement (i.e., actigraphy). Wrist-worn actigraphy measuring sleep parameters has since been validated by comparison with PSG (Kushida et al., 2001; de Souza et al., 2003; Kanady et al., 2011; Marino et al., 2013; Kosmadopoulos et al., 2014). Importantly, current practice parameters (Littner et al., 2003b) recommend actigraphy be used concurrently with the CSD to properly identify sleep windows (i.e., the period during which participants are trying to sleep). Thus, when used with the CSD, actigraphy is currently accepted as a valid, practical alternative to PSG; allowing for long-term continuous sleep assessments at home (Ancoli-Israel et al., 2003; Littner et al., 2003b). As such, actigraphy can be used as an objective measure of sleep quality in a natural environment—spanning many nights—potentially providing for better comparison with the PSQI.

The PSQI has been compared previously to actigraphy in a non-clinical sample (Grandner et al., 2006). Grandner et al. (2006) compared PSQI scores with 7 days of actigraphy and concurrent sleep diary entries in 53 younger and 59 older adults. They showed global PSQI scores did not correlate significantly with actigraphy in younger or older adults; but did correlate with sleep diary entries. These findings suggest subjective measures differ from actigraphic measures of sleep quality; however, Grandner et al. (2006) used only 7 days of actigraphy (and less than 7 days in some cases). Current recommendations for improved actigraphic assessment of sleep quality call for 14 day recordings to account for day-to-day, as well as week-to-week variability in sleep quality (VAN Someren, 2007). Perhaps longer actigraphic recordings would correlate better with the PSQI. Thus, further study is warranted to determine whether improved actigraphic estimates of sleep quality—using 14 day recordings as currently recommended—would result in better agreement between actigraphy and the PSQI.

Given the increasing number of studies testing interventions targeting sleep quality and cognitive function in older adults (Ko and Youn, 2011; Nguyen and Kruse, 2012; Schega et al., 2013; Figueiro et al., 2014; Pa et al., 2014; Richter et al., 2014; Cordi et al., 2015), we believe it is important to examine how estimates of sleep quality differ for subjective vs. objective measures—specifically for older adults. Subjective reports of sleep quality such as those provided by the PSQI may discriminate “good” vs. “poor” sleepers; however, they might not detect subtle but clinically important changes in sleep quality due to age, disease or interventions. Furthermore, while validity and reliability of self-report data are always important considerations, we propose that these psychometric issues may be especially important when using self-report measures in older adults, secondary to age-related cognitive changes in memory and executive functions. Specifically, because the PSQI requires respondents to provide answers that best reflect their sleep during the previous month, response accuracy depends at least in part on the cognitive capacity to reflect on the past month (Schacter and Addis, 2007).

Hence, the current study examined measurement and methodological issues we believe to be critical for researchers who aim to include sleep quality as an outcome measure in their studies with older adults. Our primary objective was to confirm findings from Grandner et al. (2006) using improved estimates of objective sleep quality—recording 14 days of actigraphy instead of 7 days—in accordance with current recommendations (VAN Someren, 2007). We compared sleep quality assessments for 78 community dwelling adults 55+ (mean age = 71.6) using the PSQI (Buysse et al., 1989), the CSD (Carney et al., 2012), and actigraphy (Littner et al., 2003b). Importantly, in accordance with current recommendations, the CSD was completed concurrently with actigraphy, whereas the PSQI assessed sleep quality for the month prior to actigraphy (Buysse et al., 1989; Littner et al., 2003b). This approach allowed us to assess whether potential differences between the PSQI and actigraphy are due to information source (i.e., objective vs. subjective) or due to the discrepancy between the temporality of the assessments. Our secondary objective was to extend the current science of sleep quality among older adults by determining whether observed differences in subjective vs. objective measures of sleep quality depend on age and cognitive status. Because the PSQI asks respondents to reflect on their sleep during the previous month—requiring the capacity to accurately remember one's recent past—we hypothesized that observed discrepancies between PSQI reported sleep quality and actigraphy may depend at least in part on older adults' cognitive function, as measured by standard indices of global cognitive status.

## Methods

### Participants

Community dwelling adults 55 years or older (*N* = 78) were recruited through advertising in newspapers, pamphlets distributed at local community centers, and word of mouth referrals. Upon expressing interest in participating in the study, individuals were pre-screened for eligibility criteria. We included individuals who: (1) scored ≥ 24/30 on the Mini-Mental State Exam (MMSE); and (2) were able to read, write, and speak English with acceptable visual and auditory acuity. We excluded individuals who were: (1) diagnosed with dementia of any type; (2) diagnosed with another type of neurodegenerative or neurological condition (e.g., Parkinson's disease or Multiple Sclerosis) that affects cognitive function and sleep quality; (3) planning to participate or were enrolled in a clinical drug trial; or (4) unable to speak (i.e., aphasia) as judged by an inability to communicate by phone. Individuals were not excluded based on their use of medications that affect sleep negatively or positively.

All participants provided written informed consent. Ethical approval for this study was obtained from the Vancouver Coastal Health Research Institute and the University of British Columbia's Clinical Research Ethics Board.

### Demographic variables

Participant data was collected via questionnaire to provide general information (i.e., age; gender; and education) for use in our statistical analyses. Demographic variables of interest included age, gender, and education.

### Measurement of cognitive status

We used both the MMSE (Folstein et al., 1975) and the Montreal Cognitive Assessment (MoCA; Nasreddine et al., 2005) to assess global cognitive function and identify individuals probable for mild cognitive impairment (MCI; i.e., a state of cognitive deficit that exists between normal aging and pathological decline; Reisberg et al., 1988). The MMSE is a 30-point clinical tool for grading global cognitive impairment, with lower scores indicating greater impairment. The MoCA assesses multiple domains of cognitive function, including executive functions, attention, language, memory, and orientation in a short 30-point test. It has good internal consistency and test-retest reliability, and correctly identified 90% of a large sample of individuals with MCI from two different clinics with a cut-off score of < 26/30 (Nasreddine et al., 2005). The time of day during which the MMSE and MoCA were administered varied across participants (ranging from 09:00 to 17:00 h).

### Measurement of sleep quality

#### Actigraphy

To objectively measure sleep quality, we used the MotionWatch 8© actigraphy system (MW8; cam*n*tech) a light weight, water-proof, tri-axial wrist-worn accelerometer. This improved system replaces the Actiwatch 4 and Actiwatch 7, which are discontinued. The MW8 provides reliable, previously validated estimates of daytime activity and parameters of sleep quality including sleep latency, duration, efficiency, and fragmentation (Elbaz et al., 2012; Middleton and Hampton, 2012). We used a minimum of 14 days of continuous MW8 recordings—in accordance with VAN Someren (2007)—to provide better actigraphic sleep estimates, capturing not only day-to-day variability but also week-to-week variability.

Participants were fitted with the MW8 and provided detailed information on its features (i.e., the light sensor, event marker button, and status indicator). Participants were instructed to press the event marker button each night when they started trying to sleep; and again each morning when they finished trying to sleep.

Participants were also given the Consensus Sleep Diary (CSD) and asked to complete it upon awakening each morning. After wearing the MW8 continuously for at least 14 days, participants returned the MW8 and the completed CSD. The MW8 data was subsequently downloaded and analyzed using MotionWare 1.0.27 (cam*n*tech). Responses from the CSD were used to confirm sleep windows identified by participants (as determined by time stamped event markers). In cases where the event marker and CSD entry disagreed for the start time of the sleep window, we used the light sensor data to determine “lights out.” Similarly, when the event marker and CSD entry disagreed for the end of the sleep window, we used “lights on” and activity onset to determine the end of the sleep window.

**Figure 1:**
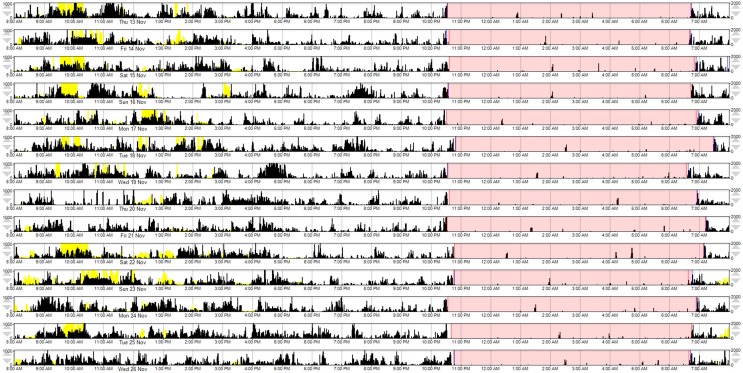
**A representative example of MW8 actigraphy for “good” sleep quality, as defined by the MW8 composite**. This actogram provides a graphical representation of the MW8 data. Each row represents a 24 h day, beginning at 8:00 a.m. The participant's “sleep window” is identified by pink shading (i.e., the period during which the participant reported they were trying to sleep). Activity counts are depicted by black vertical deflections (in counts/min; maximum visible = 1000 counts, as currently scaled). Activity during the sleep window is associated with fragmentation of sleep and awakenings. The yellow vertical deflections represent light exposure (in lux/min; maximum visible = 2000 lux, as currently scaled).

**Figure 2:**
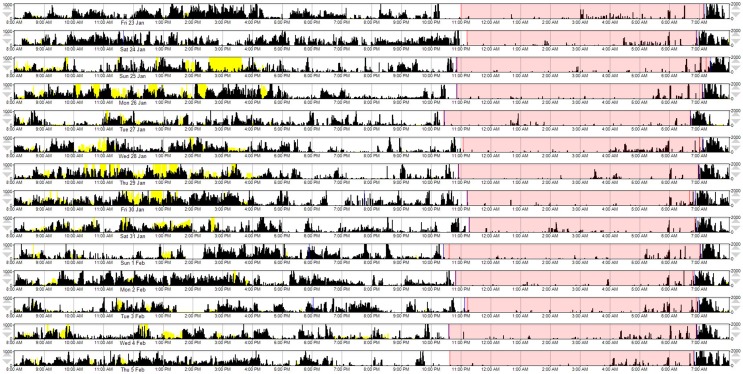
**A representative example of MW8 actigraphy for “poor” sleep quality, as defined by the MW8 composite**. This actogram provides a graphical representation of the MW8 data, with conventions as reported for Figure 1.

A composite MW8 sleep quality score was created by averaging the standardized duration, efficiency, and fragmentation scores. The fragmentation score was multiplied by −1 prior to averaging; thus, higher composite scores represent better sleep quality. Because the PSQI categorizes individuals as either “good” or “poor” quality sleepers – for comparison purposes—we created a categorical composite score for the MW8 data. In addition to considering the distribution of MW8 data in our sample, our criteria for categorizing the composite MW8 sleep quality score was based on sleep characteristics previously shown to predict cognitive decline in older adults (Keage et al., 2012). Individuals were classified as having: (1) good sleep quality based on fragmentation ≤ 25, efficiency ≥ 85, and duration ≥ 420 min (see Figure 1); (2) poor sleep quality based on fragmentation ≥ 40 and efficiency ≤ 75, or duration ≤ 360 min (see Figure 2); or (3) the remaining individuals were classified as having average sleep quality.

#### The pittsburgh sleep quality index

Subjective measurement of sleep quality for the month prior to MW8 recordings was assessed using the PSQI. The PSQI was administered on the same day they were fitted with the MW8, following completion of the MMSE and MoCA.

#### The consensus sleep diary

In addition, concurrent with the MW8 recordings, participants completed the 9-item CSD (Carney et al., 2012)—upon awakening each morning—which provided a subjective measure of sleep quality during actigraphic recordings.

## Statistical analyses

Variable distributions were initially screened for departures from normality and extreme outlying values (>3 SD from mean score). One individual had an extreme MW8 latency (48 min), 5 individuals had extreme PSQI latency values (ranging from 60 to 120 min), and one individual had an extreme sleep diary latency value (180 min). Additionally, one individual had an extreme MW8 efficiency score (57.1%). These individuals were removed from analyses involving the measure in question. The primary analyses involved bivariate and partial correlation analyses conducted using SPSS version 22 (IBM Corporation 2013). For all continuous variables, Pearson product-moment correlations were computed (r), and for correlations involving categorical variables (i.e., gender and education), Spearman's rho (ρ) was computed. Partial correlations were covaried for participant gender, age, education, MoCA score, and days of MW8 recordings. These partial correlation analyses determined whether any associations observed among the sleep quality indices could be spurious effects attributed to individual differences in demographics, cognitive status, or in MW8 wear time. In the text below, we report only the bivariate and partial correlations for between-method sleep quality comparisons because these specifically address the primary research questions. For a full report of the correlations, including within-method correlations (e.g., the correlation between MW8 fragmentation and MW8 duration) see supplementary Appendix A (bivariate correlations) and Appendix B (partial correlations).

We powered the study to detect a one-sided correlation with moderate effect size (|*r*| = 0.30) between the three methods of assessing sleep quality. Our rationale was that this effect size would be the minimal meaningful correlation desired for a subjective measure of sleep quality with an objective measure. The study sample of 78 provided approximately 87% power to detect this effect size (Faul et al., 2009).

## Results

### Participants

Descriptive statistics for the study sample are provided in Table 1. Notably, the majority of participants were female (67%), retired (92%), and had greater than a high school education (82%). One participant became ill following the 6th day of recordings—unrelated to participating in the study—rendering subsequent recordings unusable. We included this participant's 6 day recording in our analysis, following sensitivity analysis with this data excluded to confirm the data was not unduly impacted by inclusion.

**Table 1:** **Descriptive statistics for the study variables**.

**Variable**	**Mean (*SD*) or n (%)**	**Range**
Age	71.6 (6.6)	55–83
Gender, female	51 (67%)	n/a
Education, >HS diploma	62 (82%)	n/a
Employment, retired	70 (92%)	n/a
MMSE	28.8 (1.2)	25–30
MoCA	24.7 (2.6)	17–29
MW-days of wear	15.2 (2.7)	6–29
MW-latency (mins)	5.4 (4.8)	0–23
MW-efficiency	82.7 (5.8)	65.4–95.2
MW-duration (mins)	408.8 (46.4)	293–508
MW-fragmentation	31.7 (11.3)	12–73
MW-composite (z-score)	0.01 (0.85)	−2.84 to 1.72
L5 Start (hr:min)	0:40 (1:01)	23:00–3:00
M10 Start (hr:min)	8:19 (1:14)	5:00–12:00
Relative amplitude	0.89 (0.05)	0.75–0.98
Inter-daily stability	0.54 (0.11)	0.31–0.84
Intra-daily variability	0.87 (0.20)	0.47–1.47
PSQI-latency (mins)	13.5 (11.0)	2–45
PSQI-efficiency	79.6 (14.4)	44.4–100.0
PSQI-duration (mins)	376.7 (79.2)	180–570
PSQI-disturbances	10.3 (3.4)	1–17
PSQI-sleep quality	1.2 (0.9)	0–3
PSQI-total	7.4 (4.1)	1–18
PSQI sleep quality category, “good”	28 (36%)	n/a
SD-latency	22.6 (16.2)	2–72.9
SD-accuracy	91.2 (12.9)	35.7–100.0
SD-awakenings	2.1 (1.0)	0.6–5.8
SD-quality	3.4 (0.7)	2–5
SD-sleep window (mins)	461.7 (50.2)	364.3–617.5
SD-sleep duration (mins)	398.0 (61.7)	163.7–535.8

*HS, high school; MMSE, Mini-Mental State Examination; MoCA, Montreal Cognitive Assessment; MW, motion watch; PSQI, Pittsburgh Sleep Quality Index; SD, sleep diary*.

### Correlation between measures of sleep and covariates of interest

The correlations between the various sleep measures and the covariates are displayed in Table 2. Generally, the sleep indices were unrelated or were only weakly related to the covariates. Moreover, the correlations were not consistent across the three methods of quality assessment. Women tended to have later M10 start times (i.e., later start times for their 10 most active hours) and longer latencies based on their CSD entries, compared to men (ρ = 0.30 and 0.31, respectively, *p* < 0.01). Older participants tended to have greater MW8 fragmentation (*r* = 0.26, *p* < 0.05) yet reported shorter latency and fewer awakenings in their sleep diaries (*r* = –0.26 and –0.23, respectively, *p* < 0.05). Higher MoCA scores were associated with later M10 start time (*r* = 0.22, *p* < 0.05), larger relative amplitudes (*r* = 0.27, *p* < 0.05) and longer sleep latencies based on sleep diary (*r* = 0.24, *p* < 0.05). Individuals with higher educational attainment had less MW8 duration (*r* = 0.27, *p* < 0.05).

**Table 2:** **Bivariate correlations between sleep indices and covariates**.

	**Gender (female)^a^**	**Education^a^**	**Age**	**MoCA**	**Days of wear**
1. MW-latency	−0.07	−0.19	0.01	−0.05	−0.03
2. MW-efficiency	0.02	−0.15	0.07	0.06	0.11
3. MW-duration	0.05	−0.27^*^	0.01	0.08	0.07
4. MW-fragmentation	−0.19	0.08	0.26^*^	−0.22	−0.10
5. MW-composite	0.11	−0.15	−0.10	0.15	0.10
6. L5 Start	0.05	0.06	−0.03	0.03	−0.06
7. M10 Start	0.30^**^	0.09	−0.20	0.22^*^	−0.03
8. Relative amplitude	−0.03	−0.16	−0.17	0.27^*^	0.21
9. Inter-daily stability	−0.12	0.01	0.09	0.01	0.04
10. Intra-daily variability	−0.12	−0.06	0.16	−0.15	−0.07
11. PSQI-latency	0.23	0.13	−0.19	−0.07	−0.13
12. PSQI-efficiency	0.04	0.10	−0.18	−0.05	0.05
13. PSQI-duration	−0.06	−0.02	−0.17	0.01	0.01
14. PSQI-disturbances	0.21	−0.21	−0.07	0.01	−0.06
15. PSQI-sleep quality	0.17	−0.08	−0.07	0.07	−0.05
16. PSQI-total	0.17	−0.09	0.001	0.12	−0.10
17. SD-latency	0.31^**^	−0.07	−0.26^*^	0.24^*^	−0.04
18. SD-accuracy	0.15	−0.18	−0.19	0.09	0.03
19. SD-awakenings	0.08	0.17	−0.23^*^	−0.01	−0.02
20. SD-quality	−0.01	0.08	−0.004	−0.02	0.17
21. SD-sleep window	−0.14	−0.19	−0.03	−0.08	0.12
22. SD-sleep duration	−0.17	−0.05	−0.02	−0.21	0.12

a *Non-parametric Spearman's rho (ρ). MMSE, Mini-Mental State Examination; MoCA, Montreal Cognitive Assessment; MW, motion watch; PSQI, Pittsburgh Sleep Quality Index; SD, sleep diary*.

* p < 0.05;

** *p < 0.01*.

### Correlation between measures of sleep

The bivariate and partial correlations determining the associations of the MW8 and CSD sleep indices with the PSQI sleep indices are provided in Table 3 and the associations between the MW8 and CSD sleep indices are provided in Table 4. The results were highly similar between the bivariate and partial correlations; in the text, we report the partial correlations, which account for participant age, sex, education, MoCA score, and days of MW8 recordings. It is noteworthy that the covariates did not substantially alter the correlation values as this suggests that these variables, including general cognitive functioning, do not contribute to the associations between objective and subject sleep indices.

**Table 3:** **Correlations of Pittsburgh Sleep Quality Index measures with MotionWatch and Sleep Diary Measures**.

	**PSQI measures**					
	**Latency**	**Efficiency**	**Duration**	**Disturbances**	**Sleep quality**	**Total**
MW-latency	0.21/0.22	0.03/0.05	0.04/0.03	−0.02/−0.08	0.02/−0.01	0.08/0.05
MW-efficiency	−0.11/−0.08	−0.03/0.002	0.07/0.10	0.08/0.08	−0.18/−0.20	−0.09/−0.12
MW-duration	0.12/0.16	−0.11/−0.07	0.29^**^/0.32^**^	0.23^*^/0.18	−0.10/−0.15	0.03/−0.02
MW-fragmentation	0.05/0.12	−0.01/0.000	0.02/0.04	−0.03/0.05	0.05/0.12	0.02/0.08
MW-composite	−0.004/−0.02	−0.05/−0.03	0.10/0.11	0.14/0.08	−0.09/−0.15	−0.01/−0.07
L5 Start	0.000/−0.02	0.24^*^/0.24^*^	0.26^*^/0.26^*^	−0.10/−0.11	−0.31^**^/−0.33^**^	−0.21/−0.24^*^
M10 Start	0.03/−0.03	0.19/0.21	0.15/0.16	−0.01/−0.07	−0.05/−0.11	−0.07/−0.15
Relative amplitude	−0.03/0.02	−0.01/−0.01	0.13/0.11	−0.11/−0.12	−0.16/−0.19	−0.07/−0.08
Inter-daily stability	−0.08/−0.02	−0.22/−0.22	−0.12/−0.13	−0.05/0.01	0.09/0.14	0.09/0.13
Intra-daily variability	0.03/0.05	−0.01/0.01	−0.06/−0.05	−0.04/−0.03	−0.02/0.001	−0.02/−0.01
SD-latency	0.56^***^/0.56^***^	−0.45^***^/−0.51^***^	−0.22/−0.25^*^	0.18/0.11	0.29^*^/0.23	0.52^***^/0.49^***^
SD-accuracy	−0.07/−0.14	−0.05/−0.06	0.21/0.21	0.18/0.11	0.02/−0.04	−0.02/−0.08
SD-awakenings	0.13/0.06	−0.11/−0.20	0.06/0.02	0.09/0.11	0.11/0.12	0.04/0.08
SD-quality	−0.24^*^/−0.24^*^	0.43^***^/0.43^***^	0.40^***^/.43^***^	−0.37^**^/−0.36^**^	−0.64^***^/−0.65^***^	−0.56^***^/−0.55^***^
SD-sleep window	0.11/0.14	0.05/0.05	0.53^***^/0.54^***^	0.21/0.21	−0.06/−0.06	−0.09/−0.08
SD-sleep duration	−0.12/−0.14	0.45^***^/0.46^***^	0.72^***^/0.75^***^	0.03/0.04	−0.38^***^/−0.40^***^	−0.48^***^/−0.48^***^

*Bivariate correlations appear on the left side of the forward slash and partial correlations appear on the right side. Partial correlations are covaried for participant gender, age, Montreal Cognitive Assessment score, education, and days of Motion Watch wear. MW, motion watch; PSQI, Pittsburgh Sleep Quality Index; SD, sleep diary*.

* p < 0.05;

** p < 0.01;

*** *p < 0.001*.

**Table 4:** **Correlations of Sleep Diary with MotionWatch Measures**.

	**SD measures**					
	**Latency**	**Accuracy**	**Awakenings**	**Quality**	**Sleep window**	**Sleep duration**
MW-latency	0.31^**^/0.33^**^	−0.18/−0.22	−0.001/0.02	0.02/0.05	0.05/0.01	−0.07/−0.11
MW-efficiency	−0.09/−0.10	0.30^**^/0.31^**^	−0.07/−.03	0.19/0.19	0.05/0.03	0.14/0.15
MW-duration	0.16/0.14	0.30^**^/0.27^*^	0.03/0.08	0.08/0.11	0.61^***^/0.61^***^	0.45^***^/0.49^***^
MW-fragmentation	0.01/0.15	−0.24^*^/−0.17	0.06/0.11	−0.17/−0.19	0.15/0.16	0.01/−0.02
MW-composite	0.01/−0.07	0.30^**^/0.26^*^	−0.06/−0.06	0.13/0.16	0.13/0.11	0.20/0.23
L5 Start	−0.02/−0.05	−0.07/−0.08	0.13/0.13	0.21/0.23	0.26^*^/0.29^*^	0.29^**^/0.34^**^
M10 Start	0.000/−0.14	−0.14/−0.21	0.07/0.03	0.06/0.08	0.05/0.11	0.13/0.23
Relative amplitude	0.14/0.11	0.21/0.19	−0.06/−0.06	0.17/0.18	−0.01/−0.04	−0.01/−0.001
Inter-daily stability	0.12/0.20	−0.01/0.03	0.19/0.23	−0.16/−0.18	0.01/0.01	−0.28^*^/−0.30^**^
Intra-daily variability	−0.28^*^/−0.24^*^	−0.05/−0.02	−0.05/−0.02	−0.05/−0.05	−0.06/−0.08	0.06/0.04

*Bivariate correlations appear on the left side of the forward slash and partial correlations appear on the right side. Partial correlations are covaried for participant gender, age, Montreal Cognitive Assessment score, education, and days of Motion Watch wear. MW, motion watch; PSQI, Pittsburgh Sleep Quality Index; SD, sleep diary*.

* p < 0.05;

** p < 0.01;

*** *p < 0.001*.

Although there were many instances in which sleep indices were moderately or strongly correlated with other sleep indices using the same measurement method (especially MW8; please see supplementary Appendices A, B for details), correlations across the three methods were generally weak or non-significant, especially between MW8 and the two subjective methods. MW8 duration correlated modestly with PSQI duration (partial *r* = 0.32, *p* < 0.01) but more strongly with CSD duration (partial *r* = 0.49, *p* < 0.001). CSD duration was strongly related to PSQI duration (partial *r* = 0.75, *p* < 0.001). Moreover, the CSD sleep quality score was fairly strongly associated with the PSQI sleep quality score (partial *r* = −0.65, *p* < 0.001). The MW8 composite score was unrelated to any PSQI score, but was related to the CSD accuracy score (partial *r* = 0.26, *p* < 0.05). The CSD accuracy score (defined by CSD sleep window agreement with MW8 event markers, actigraphy, and light recordings) was also associated with the MW8 efficiency score (partial *r* = 0.31, *p* < 0.01) and the MW8 duration score (partial *r* = 0.27, *p* < 0.05).

### Categorizing sleep quality: “good” vs. “poor” sleepers

Next, we compared the MW8-based sleep categorization to the PSQI-based sleep categorization. The cross-tabulation is shown in Table 5, which reveals that individuals categorized as poor sleepers based on the PSQI were equally likely to be classified as poor or good sleepers based on the MW8 data. Similarly, good sleepers based on the PSQI were equally likely to be classified as poor or good sleepers based on the MW8 data. The chi-square test was not significant (*p* = 0.907), which confirms that categorization based on the PSQI was unrelated to categorization based on the MW8. We then classified individuals based on the nature of the discrepancy between the PSQI sleep quality score and the MW8 sleep quality score. Individuals were classified as “under-estimators” (i.e., PSQI category was “poor” but the MW8 category was “average” or “good”; *n* = 39), “accurate” (i.e., PSQI category matched the MW8 category; *n* = 15), or as “over-estimators” (i.e., PSQI category was “good” but the MW8 category was “average” or “poor”; *n* = 23). We then examined differences in the demographic and cognition variables based on this categorization; as shown in Table 6, there were no significant differences among these three groups. Thus, these variables do not explain the discrepancy between objective and subjective reports of sleep quality.

**Table 5:** **Cross-tabulation of MotionWatch-categorized sleep quality vs. PSQI-categorized sleep quality**.

		**PSQI sleep category**		
		**Poor**	**Good**	**Total**
MotionWatch sleep category	Poor	10	5	15
	Average	29	18	47
	Good	10	5	15
	Total	49	28	77

*χ^2^ (2) = 0.195, p = 0.907*.

**Table 6:** **Comparison of accurate and inaccurate self-reports on the PSQI on demographics and cognition**.

**Variable**	**Underestimate on PSQI *n* = 39**	**Accurate on PSQI *n* = 15**	**Overestimate on PSQI *n* = 23**	
	**Mean (*SD*) or n (%)**	**Mean (*SD*) or n (%)**	**Mean (*SD*) or n (%)**	***p*-value^a^**
Age	71.7 (6.7)	71.9 (6.4)	71.7 (6.7)	0.992
Gender, female	28 (72%)	10 (67%)	13 (57%)	0.470
Education, >HS diploma	30 (77%)	13 (87%)	20 (87%)	0.529
Employment, retired	37 (95%)	14 (93%)	20 (87%)	0.524
MMSE	28.9 (1.0)	28.2 (1.9)	29.0 (1.1)	0.122
MoCA	25.0 (2.4)	23.9 (3.2)	24.9 (2.7)	0.433
MW-days of wear	14.9 (2.6)	15.3 (2.6)	15.7 (3.1)	0.564

a *p-value comes from One-Way ANOVA (continuous variables) or χ^2^ test (categorical variables). HS, high school; MMSE, Mini-Modified State Examination; MoCA, Montreal Cognitive Assessment; PSQI, Pittsburgh Sleep Quality Index*.

### Sensitivity analyses

In a set of sensitivity analyses, we reran the above analyses excluding the one individual who had less than 14 days of MW8 wear. All significant correlations were replicated in these analyses (not shown). In a second set of sensitivity analyses, we reran the primary bivariate and partial correlation analyses including the extreme outlier scores, which can be found in see supplementary Appendices C, D, respectively. The correlation values did not differ from those reported above, based on their statistical comparison using Fisher's r-to-z transformation.

## Discussion

Sleep changes with aging, and thus, sleep complaints are common among older adults. Recent findings suggest sleep quality plays a critical role in preserving cognitive function in older adults and reducing the risk of AD (Lim et al., 2013)—the most common cause of dementia. Therefore, understanding age-related changes in sleep and their potential to impact cognitive function in older adults has become a research priority. However, sleep quality is a complex construct to evaluate empirically. We believe the validity of future research efforts depends greatly on the methods used to quantify parameters of sleep quality. Subjective sleep quality measures such as the PSQI are quick and easy to use, and thus, these measures are commonly used in intervention studies and randomized controlled trials (Ko and Youn, 2011; Nguyen and Kruse, 2012; Schega et al., 2013; Figueiro et al., 2014; Pa et al., 2014; Richter et al., 2014). In fact, for many studies, the PSQI is the only measure used to quantify sleep quality. Importantly, the current study's findings suggest subjective measures of sleep quality (i.e., the PSQI and the CSD) vs. objective measures (i.e., actigraphy) survey different aspects of sleep quality. It would seem—for older adults at least—perceived sleep quality is something quite different from objective sleep quality.

When considered in combination with earlier findings (i.e., Grandner et al., 2006; and the original PSQI validation paper showing lack of agreement between the PSQI and PSG, Buysse et al., 1989), our results provide further support suggesting differences in sleep quality as measured by the PSQI vs. objective measures are likely not just the result of temporal differences in the recording period. We compared actigraphy spanning at least 14 days (with one exception as noted) and still none of our objective measures of sleep quality (i.e., duration, efficiency, and fragmentation) correlated with global PSQI scores. Because the PSQI was originally designed to categorize people as “good” vs. “poor” sleepers; for better comparison, we then created a composite MW8 sleep quality score to classify sleepers (by averaging the standardized duration, efficiency, and fragmentation scores). Remarkably—as shown in Table 5—PSQI classification as either a “good” or a “poor” sleeper provides no predictive validity in determining an individual's objective sleep quality, as measured by MW8 actigraphy.

This lack of PSQI predictive validity for actigraphy-defined sleep quality might be explained by a temporal difference in sleep quality from the month surveyed by the PSQI and the 14 days of actigraphy that followed. Interestingly, the CSD defined sleep duration and quality were strongly associated with PSQI defined sleep duration and quality, despite the lack of temporal overlap. These results suggest subjective sleep quality did not change significantly from the month preceding MW8 recordings to the MW8 recording period. The CSD defined duration also correlated with MW8 duration, but this correlation was smaller in size compared to those between CSD and PSQI. Finally, the correlation between MW8 duration and PSQI duration was only moderate in size. Taken together, these findings suggest the temporal difference between PSQI and actigraphy might explain their discrepancies in part, but a stronger contributor is the subjective vs. objective distinction.

Age-related changes in memory and executive function—resulting in systematic differences in PSQI response accuracy—could also explain discrepancies we observed between PSQI and actigraphy defined sleep quality. However, our results suggest neither age, nor cognitive status—as defined by MMSE and MoCA scores—explain the difference between subjective and objective measures of sleep quality. This conclusion comes from our analysis of “under-estimators” (i.e., individuals whose PSQI defined sleep quality was poor but whose MW8 defined sleep quality was average or good), “accurate estimators” (i.e., individuals whose PSQI defined sleep quality matched their MW8 defined sleep quality), and “over-estimators” (i.e., individuals whose PSQI defined sleep quality was good but whose MW8 defined sleep quality was poor or average). Interestingly, more individuals were “under-estimators” (*n* = 39; 51% of sample) compared to “over-estimators” (*n* = 23, 30% of sample). This suggests that older adults might tend to perceive their sleep as worse than it actually might be. When we compared these three categories of sleep estimation on a variety of demographic and cognitive variables, we found no differences between groups on any of these variables. Thus, the contributing factors to an older individual underestimating vs. overestimating her sleep remain elusive. However, previous findings have shown patients with insomnia not only underestimate sleep duration but also overestimate sleep latency (Frankel et al., 1976). In this respect, over half of the older adults we observed were similar to patients with insomnia. Importantly, this finding suggests—when studying older adults—subjective measures of sleep quality should be used with caution.

It is important to note that in the few instances in which there was a significant correlation between an objective and subjective sleep measure (as detailed in Tables 3, 4), the correlation value was largely unaffected when controlling for cognitive performance and demographic variables. This finding provides confidence that these correlations reflect true associations rather than spurious ones driven by a third variable (e.g., cognitive performance or age). It is also noteworthy how few of the correlations between subjective and objective sleep measures were significant, especially between the PSQI and MW8. Of the 60 correlations comparing these two measures (10 MW8 variables by 6 PSQI variables), only five were significant in the partial correlation analysis (see Table 3), of which three would be expected by chance (60 multiplied an alpha of 0.05).

Taken together, our findings suggest—at least for older adults—subjective measures of sleep quality (i.e., the PSQI and CSD) survey different aspects of sleep quality, when compared with objective measures (i.e., actigraphy). Based on our data, it would seem an older adult's perception of their sleep quality is quite different from objective reality. As such, we conclude the PSQI does not provide predictive validity for an older adult's objective sleep quality; and thus, should be used with caution.

However, we in no way mean to suggest PSQI reported sleep quality is unimportant. In fact, many studies have shown PSQI scores correlate with diseases of aging and mortality (Martin et al., 2011; Kang et al., 2012; Gao et al., 2014; Mellor et al., 2014; Zhang et al., 2014; Lajoie et al., 2015; Lou et al., 2015). Clearly, PSQI scores reflect an important aspect of sleep quality, as is true for objective measures. However, because subjective vs. objective measures appear to target very different aspects of sleep quality—each providing valuable insight—we recommend best practice would be include both subjective and objective measures when examining sleep quality in older adults (i.e., the PSQI, CSD, and actigraphy).

We acknowledge it is not always practical to include both subjective and objective measures of sleep quality; in large epidemiological studies for example. Thus, we believe further study to identify factors explaining why for some, perception more closely reflects objective reality, whereas for many it does not. Perhaps a PSQI correction factor could eventually be devised, providing significantly improved predictive validity of objective sleep quality.

## Limitations

Our findings are limited by the typical issues of generalizability, such that our conclusions apply specifically to healthy community-dwelling adults aged 55 years or older. Related, the sample was not gender balanced, as approximately two-thirds of participants were female. In addition, our comparisons between the PSQI and MW8 actigraphy are limited by temporal differences in the recording period. As discussed in the text, the PSQI surveys sleep quality for the month prior to the assessment session; whereas, MW8 recordings started at 8:00 a.m the morning following the assessment session. Participants began completing CSD entries each morning upon awakening following the first night of MW8 recordings. Scheduling the PSQI in advance of MW8 recordings was deemed necessary so as not to contaminate PSQI responses. Our concern was that the process of completing the CSD upon awakening each morning would enhance PSQI response accuracy. However, subjective sleep quality does not appear to have changed significantly from the month preceding MW8 recordings, to the MW8 recording period, as evidenced by the strong correlation observed between CSD and PSQI reported sleep quality. Finally, we note that a large number of correlation analyses were run, which inflates the type II error rate. Nevertheless, the general conclusion we draw from these analyses is that very few between-method correlations were significant in spite of the fact that many correlations were computed, and thus, these varying methods of assessing sleep quality are largely unrelated.

### Conflict of interest statement

The authors declare that the research was conducted in the absence of any commercial or financial relationships that could be construed as a potential conflict of interest.
