# Supplementary material for: Measuring sleep quality in older adults: a comparison using subjective and objective methods
Source: Front Aging Neurosci. 2015 Sep 7;7:166. doi: 10.3389/fnagi.2015.00166 (PMC4561455; doi:10.3389/fnagi.2015.00166)
Supplement: Supplementary file 1 [file AppendixA.DOCX]

**Appendix A**. Bivariate correlations among sleep quality indices.

|  | 1. | 2. | 3. | 4. | 5. | 6. | 7. |
| --- | --- | --- | --- | --- | --- | --- | --- |
| 1. MW-latency | 1.00 |  |  |  |  |  |  |
| 2. MW-efficiency | -.27* | 1.00 |  |  |  |  |  |
| 3. MW-duration | -.15 | .56*** | 1.00 |  |  |  |  |
| 4. MW-fragmentation | .12 | -.71*** | -.41*** | 1.00 |  |  |  |
| 5. MW-composite | -.23* | .89*** | .77*** | -.86*** | 1.00 |  |  |
| 6. L5 Start | -.002 | -.23* | -.05 | .22 | -.21 | 1.00 |  |
| 7. M10 Start | .09 | -.22 | -.07 | .05 | -.12 | .54*** | 1.00 |
| 8. Relative Amplitude | -.13 | .51*** | .48*** | -.49*** | .61*** | -.26* | -.24* |
| 9. Inter-daily Stability | -.05 | -.04 | -.03 | .11 | -.09 | -.17 | -.27* |
| 10. Intra-daily Variability | -.08 | .10 | -.11 | -.02 | -.004 | .01 | -.02 |
| 11. PSQI-latency | .21 | -.11 | .12 | .05 | -.004 | .000 | .03 |
| 12. PSQI-efficiency | .03 | -.03 | -.11 | -.01 | -.05 | .24* | .19 |
| 13. PSQI-duration | .04 | .07 | .29** | .02 | .10 | .26* | .15 |
| 14. PSQI-disturbances | -.02 | .08 | .23* | -.03 | .14 | -.10 | -.01 |
| 15. PSQI-sleep quality | .02 | -.18 | -.10 | .05 | -.09 | -.31** | -.05 |
| 16. PSQI-total | .08 | -.09 | .03 | .02 | -.01 | -.21 | -.07 |
| 17. SD-latency | .31** | -.09 | .16 | .01 | .01 | -.02 | .000 |
| 18. SD-accuracy | -.18 | .30** | .30** | -.24* | .30** | -.07 | -.14 |
| 19. SD-awakenings | -.001 | -.07 | .03 | .06 | -.06 | .13 | .07 |
| 20. SD-quality | .02 | .19 | .08 | -.17 | .13 | .21 | .06 |
| 21. SD-sleep window | .05 | .05 | .61*** | .15 | .13 | .26* | .05 |
| 22. SD-sleep duration | -.07 | .14 | .45*** | .01 | .20 | .29** | .13 |

Abbreviations: MW = motion watch. PSQI = Pittsburgh Sleep Quality Index. SD = sleep diary.

**p* < .05. ** *p* < .01. *** *p* < .001.

|  | 8. | 9. | 10. | 11. | 12. | 13. | 14. | 15. |
| --- | --- | --- | --- | --- | --- | --- | --- | --- |
| 1. MW-latency |  |  |  |  |  |  |  |  |
| 2. MW-efficiency |  |  |  |  |  |  |  |  |
| 3. MW-duration |  |  |  |  |  |  |  |  |
| 4. MW-fragmentation |  |  |  |  |  |  |  |  |
| 5. MW-composite |  |  |  |  |  |  |  |  |
| 6. L5 Start |  |  |  |  |  |  |  |  |
| 7. M10 Start |  |  |  |  |  |  |  |  |
| 8. Relative Amplitude | 1.00 |  |  |  |  |  |  |  |
| 9. Inter-daily Stability | .33** | 1.00 |  |  |  |  |  |  |
| 10. Intra-daily Variability | -.42*** | -.53*** | 1.00 |  |  |  |  |  |
| 11. PSQI-latency | -.03 | -.08 | .03 | 1.00 |  |  |  |  |
| 12. PSQI-efficiency | -.01 | -.22 | -.01 | -.22 | 1.00 |  |  |  |
| 13. PSQI-duration | .13 | -.12 | -.06 | .000 | .74*** | 1.00 |  |  |
| 14. PSQI-disturbances | -.11 | -.05 | -.04 | .21 | -.25* | -.23* | 1.00 |  |
| 15. PSQI-sleep quality | -.16 | .09 | -.02 | .22 | -.61*** | -.54*** | .44*** | 1.00 |
| 16. PSQI-total | -.07 | .09 | -.02 | .40*** | -.80*** | -.68*** | .50*** | .79*** |
| 17. SD-latency | .14 | .12 | -.28* | .56*** | -.45*** | -.22 | .18 | .29* |
| 18. SD-accuracy | .21 | -.01 | -.05 | -.07 | -.05 | .21 | .18 | .02 |
| 19. SD-awakenings | -.06 | .19 | -.05 | .13 | -.11 | .06 | .09 | .11 |
| 20. SD-quality | .17 | -.16 | -.05 | -.24* | .43*** | .40*** | -.37** | -.64*** |
| 21. SD-sleep window | -.01 | .01 | -.06 | .11 | .05 | .53*** | .21 | -.06 |
| 22. SD-sleep duration | -.01 | -.28* | .06 | -.12 | .45*** | .72*** | .03 | -.38*** |

**Appendix A** (continued).

Abbreviations: MW = motion watch. PSQI = Pittsburgh Sleep Quality Index. SD = sleep diary.

**p* < .05. ** *p* < .01. *** *p* < .001.

**Appendix A** (continued).

|  | 16. | 17. | 18. | 19. | 20. | 21. | 22. |
| --- | --- | --- | --- | --- | --- | --- | --- |
| 1. MW-latency |  |  |  |  |  |  |  |
| 2. MW-efficiency |  |  |  |  |  |  |  |
| 3. MW-duration |  |  |  |  |  |  |  |
| 4. MW-fragmentation |  |  |  |  |  |  |  |
| 5. MW-composite |  |  |  |  |  |  |  |
| 6. L5 Start |  |  |  |  |  |  |  |
| 7. M10 Start |  |  |  |  |  |  |  |
| 8. Relative Amplitude |  |  |  |  |  |  |  |
| 9. Inter-daily Stability |  |  |  |  |  |  |  |
| 10. Intra-daily Variability |  |  |  |  |  |  |  |
| 11. PSQI-latency |  |  |  |  |  |  |  |
| 12. PSQI-efficiency |  |  |  |  |  |  |  |
| 13. PSQI-duration |  |  |  |  |  |  |  |
| 14. PSQI-disturbances |  |  |  |  |  |  |  |
| 15. PSQI-sleep quality |  |  |  |  |  |  |  |
| 16. PSQI-total | 1.00 |  |  |  |  |  |  |
| 17. SD-latency | .52*** | 1.00 |  |  |  |  |  |
| 18. SD-accuracy | -.02 | .05 | 1.00 |  |  |  |  |
| 19. SD-awakenings | .04 | -.01 | .20 | 1.00 |  |  |  |
| 20. SD-quality | -.56*** | -.23* | -.01 | -.20 | 1.00 |  |  |
| 21. SD-sleep window | -.09 | .15 | .26* | .17 | .08 | 1.00 |  |
| 22. SD-sleep duration | -.48*** | -.27* | .20 | .03 | .40*** | .72*** | 1.00 |

Abbreviations: MW = motion watch. PSQI = Pittsburgh Sleep Quality Index. SD = sleep diary.

**p* < .05. ** *p* < .01. *** *p* < .001.
