# Supplementary material for: Measuring sleep quality in older adults: a comparison using subjective and objective methods
Source: Front Aging Neurosci. 2015 Sep 7;7:166. doi: 10.3389/fnagi.2015.00166 (PMC4561455; doi:10.3389/fnagi.2015.00166)
Supplement: Supplementary file 4 [file AppendixD.DOCX]

**Appendix D**. Partial correlations among sleep quality indices with no outlier exclusions.

|  | 1. | 2. | 3. | 4. | 5. | 6. | 7. |
| --- | --- | --- | --- | --- | --- | --- | --- |
| 1. MW-latency | 1.00 |  |  |  |  |  |  |
| 2. MW-efficiency | -.47*** | 1.00 |  |  |  |  |  |
| 3. MW-duration | -.20 | .53*** | 1.00 |  |  |  |  |
| 4. MW-fragmentation | .26* | -.79*** | -.42*** | 1.00 |  |  |  |
| 5. MW-composite | -.37** | .91*** | .77*** | -.87*** | 1.00 |  |  |
| 6. L5 Start | .02 | -.27* | -.04 | .24* | -.22 | 1.00 |  |
| 7. M10 Start | .04 | -.21 | -.08 | .16 | -.18 | .55*** | 1.00 |
| 8. Relative Amplitude | -.19 | .59*** | .49*** | -.49*** | .62*** | -.28* | -.30** |
| 9. Inter-daily Stability | -.08 | -.09 | -.03 | .07 | -.07 | -.16 | -.25* |
| 10. Intra-daily Variability | -.05 | .09 | -.11 | -.09 | .02 | .02 | .03 |
| 11. PSQI-latency | .03 | -.04 | .09 | .06 | -.001 | .01 | -.06 |
| 12. PSQI-efficiency | -.07 | -.01 | -.07 | .000 | -.03 | .24* | .21 |
| 13. PSQI-duration | -.02 | -.01 | .32** | .04 | .11 | .26* | .16 |
| 14. PSQI-disturbances | .01 | .07 | .18 | .05 | .08 | -.11 | -.07 |
| 15. PSQI-sleep quality | -.05 | -.12 | -.15 | .12 | -.15 | -.33** | -.11 |
| 16. PSQI-total | .10 | -.07 | -.02 | .08 | -.07 | -.24* | -.15 |
| 17. SD-latency | .16 | -.19 | .15 | .22 | -.10 | .09 | -.04 |
| 18. SD-accuracy | -.12 | .21 | .27* | -.17 | .26* | -.08 | -.21 |
| 19. SD-awakenings | .09 | -.11 | .08 | .11 | -.06 | .13 | .03 |
| 20. SD-quality | .04 | .10 | .11 | -.19 | .16 | .23 | .08 |
| 21. SD-sleep window | .09 | -.15 | .61*** | .16 | .11 | .29* | .11 |
| 22. SD-sleep duration | -.02 | .08 | .49*** | -.02 | .23 | .34** | .23 |

Correlations are covaried for participant gender, age, Montreal Cognitive Assessment score, education, and days of Motion Watch wear. Abbreviations: MW = motion watch. PSQI = Pittsburgh Sleep Quality Index. SD = sleep diary.

**p* < .05. ** *p* < .01. *** *p* < .001.

**Appendix D** (continued).

|  | 8. | 9. | 10. | 11. | 12. | 13. | 14. | 15. |
| --- | --- | --- | --- | --- | --- | --- | --- | --- |
| 1. MW-latency |  |  |  |  |  |  |  |  |
| 2. MW-efficiency |  |  |  |  |  |  |  |  |
| 3. MW-duration |  |  |  |  |  |  |  |  |
| 4. MW-fragmentation |  |  |  |  |  |  |  |  |
| 5. MW-composite |  |  |  |  |  |  |  |  |
| 6. L5 Start |  |  |  |  |  |  |  |  |
| 7. M10 Start |  |  |  |  |  |  |  |  |
| 8. Relative Amplitude | 1.00 |  |  |  |  |  |  |  |
| 9. Inter-daily Stability | .34** | 1.00 |  |  |  |  |  |  |
| 10. Intra-daily Variability | -.41*** | -.56*** | 1.00 |  |  |  |  |  |
| 11. PSQI-latency | .12 | .06 | -.07 | 1.00 |  |  |  |  |
| 12. PSQI-efficiency | -.01 | -.22 | .01 | -.37** | 1.00 |  |  |  |
| 13. PSQI-duration | .11 | -.13 | -.05 | -.16 | .74*** | 1.00 |  |  |
| 14. PSQI-disturbances | -.12 | .01 | -.03 | .16 | -.24* | -.24* | 1.00 |  |
| 15. PSQI-sleep quality | -.19 | .14 | .001 | .30* | -.62*** | -.56*** | .39*** | 1.00 |
| 16. PSQI-total | -.08 | .13 | -.01 | .50*** | -.81*** | -.70*** | .46*** | .78*** |
| 17. SD-latency | .07 | .21 | -.21 | .41*** | -.38** | -.16 | .14 | .30* |
| 18. SD-accuracy | .19 | .03 | -.02 | -.04 | -.06 | .21 | .11 | -.04 |
| 19. SD-awakenings | -.06 | .23 | -.02 | -.09 | -.20 | .02 | .11 | .12 |
| 20. SD-quality | .18 | -.18 | -.05 | -.07 | .43*** | .43*** | -.36** | -.65*** |
| 21. SD-sleep window | -.04 | .01 | -.08 | .06 | .05 | .54*** | .21 | -.06 |
| 22. SD-sleep duration | -.001 | -.30** | .04 | -.14 | .46*** | .75*** | .04 | -.40*** |

Correlations are covaried for participant gender, age, Montreal Cognitive Assessment score, and days of Motion Watch wear.

Abbreviations: MW = motion watch. PSQI = Pittsburgh Sleep Quality Index. SD = sleep diary.

**p* < .05. ** *p* < .01. *** *p* < .001.

**Appendix D** (continued).

|  | 16. | 17. | 18. | 19. | 20. | 21. | 22. |
| --- | --- | --- | --- | --- | --- | --- | --- |
| 1. MW-latency |  |  |  |  |  |  |  |
| 2. MW-efficiency |  |  |  |  |  |  |  |
| 3. MW-duration |  |  |  |  |  |  |  |
| 4. MW-fragmentation |  |  |  |  |  |  |  |
| 5. MW-composite |  |  |  |  |  |  |  |
| 6. L5 Start |  |  |  |  |  |  |  |
| 7. M10 Start |  |  |  |  |  |  |  |
| 8. Relative Amplitude |  |  |  |  |  |  |  |
| 9. Inter-daily Stability |  |  |  |  |  |  |  |
| 10. Intra-daily Variability |  |  |  |  |  |  |  |
| 11. PSQI-latency |  |  |  |  |  |  |  |
| 12. PSQI-efficiency |  |  |  |  |  |  |  |
| 13. PSQI-duration |  |  |  |  |  |  |  |
| 14. PSQI-disturbances |  |  |  |  |  |  |  |
| 15. PSQI-sleep quality |  |  |  |  |  |  |  |
| 16. PSQI-total | 1.00 |  |  |  |  |  |  |
| 17. SD-latency | .46*** | 1.00 |  |  |  |  |  |
| 18. SD-accuracy | -.08 | .01 | 1.00 |  |  |  |  |
| 19. SD-awakenings | .08 | .04 | .20 | 1.00 |  |  |  |
| 20. SD-quality | -.55*** | -.22 | .02 | -.22 | 1.00 |  |  |
| 21. SD-sleep window | -.08 | .20 | .24* | .20 | .08 | 1.00 |  |
| 22. SD-sleep duration | -.48*** | -.19 | .21 | .02 | .41*** | .71*** | 1.00 |

Correlations are covaried for participant gender, age, Montreal Cognitive Assessment score, and days of Motion Watch wear.

Abbreviations: MW = motion watch. PSQI = Pittsburgh Sleep Quality Index. SD = sleep diary.

**p* < .05. ** *p* < .01. *** *p* < .001.
